# Syncytiotrophoblast-Derived Extracellular Vesicles in Pathophysiology of Preeclampsia

**DOI:** 10.3389/fphys.2019.01236

**Published:** 2019-10-01

**Authors:** Cha Han, Lulu Han, Pengzhu Huang, Yuanyuan Chen, Yingmei Wang, Fengxia Xue

**Affiliations:** Department of Obstetrics and Gynecology, Tianjin Medical University General Hospital, Tianjin, China

**Keywords:** preeclampsia, extracellular vesicles, inflammation, endothelial dysfunction, coagulation dysregulation

## Abstract

Preeclampsia is a common obstetric complication associated with pregnancy and it endangers lives of the mother and the infant. The histopathological changes associated with preeclampsia include systemic endothelial dysfunction, persistent inflammatory state, and coagulation and fibrinolysis dysregulations. Preeclampsia is considered to be caused by the systemic vasoconstriction of small arteries and disruption of the endothelial integrity, resulting in hypertension, proteinuria, and multiple organ dysfunction. However, mediators that trigger or propagate the pathology of preeclampsia remain poorly defined. Syncytiotrophoblast-derived extracellular vesicles (SDEVs) are increasingly recognized as a key mediator for the development of preeclampsia, but the underlying mechanisms through which these SDEVs are released and induce systemic responses are not fully understood. This review focuses on multiple roles of SDEVs in the pathogenesis of preeclampsia.

## Introduction

Preeclampsia (PE) is a pregnancy-associated pathology that is characterized by poor placentation and endothelial dysfunction (Shan et al., 2015). The prevalence of PE is reported to be 2 to 5% in the United States (Ananth et al., 2013), but is 9.3% in some areas of Africa (Abalos et al., 2013). It often results in severe complications that require early termination of the pregnancy. The rate of severe PE in the United States increased from 0.3% in 1980 to 1.4% in 2010 (Ananth et al., 2013). More importantly, pregnant women delivering in 2003 had 6.7-fold increased risk of severe pre-eclampsia, as compared to those delivering in 1980 (Ananth et al., 2013). This increase in severe PE is attributed to the age-period-cohort effects, but also suggest no significant improvements in the presentation and treatment of PE, despite significant advances in woman health and maternity care during this period. Severe PE accounted for 40% of patients with hypertensive disorders of pregnancy, while mild PE accounted for 15.1% in a Chinese survey (Ye et al., 2014).

Preeclampsia is clinically defined by newly onset hypertension (systolic and diastolic blood pressures of ≥140 and ≥90 mmHg, respectively), proteinuria (≥ 300 mg over 24 h), and organ dysfunction after 20 weeks of gestation (Chaiworapongsa et al., 2014). It carries a significant risk of progression to eclampsia, in which PE patients develop convulsions, resulting in multiple complications and maternal and fetal death (American College of Obstetricians and Gynecologists, and Task Force on Hypertension in Pregnant, 2013). Despite its association with severe clinical consequences, PE remains a major challenge in the clinical management of pregnant women (Tranquilli et al., 2014; Phoa et al., 2016), largely due to the poor understanding of its pathogenesis and causal factors. During normal pregnancy, trophoblasts infiltrate the placenta spiral artery wall to gradually replace the vascular endothelium in a process called vascular remodeling (Skow et al., 2017). The inhibition or dysregulation of this remodeling process would result in placental ischemia and reperfusion injury (Chaiworapongsa et al., 2014; Amaral et al., 2017). For example, poor placental vascular remodeling results in the formation of abnormally narrow spiral arterioles, which reduces placental blood flow (Lee et al., 2012), leading to tissue ischemia, endothelial injury, microvascular thrombosis, and inflammation (Roland et al., 2016). The degree of trophoblastic invasion defects is associated with the severity of hypertension in patients with PE (Madazli et al., 2000; Velicky et al., 2018).

A longstanding question has been how these placental pathologies are disseminated systemically to cause hypertension, proteinuria, and organ dysfunction. Many factors have been investigated, including extracellular vesicles (EVs), especially those from injured placentas. Although the placenta contains mesenchymal cells, fibroblasts within villous core stroma, and vascular cells (i.e., smooth muscle cells, pericytes, and endothelial cells), syncytiotrophoblasts are the primary cells that form the outer syncytial layer of the trophoblasts and actively invade the uterine wall to form the outermost fetal layer of the placenta. These cells cover the entire surface of the villous tree, are therefore exposed to the maternal circulation, which provides essential nutrients and allows gas exchange between the maternal and fetal circulations. Because of their location and large numbers, these apoptotic syncytiotrophoblast cells formed during placental ischemia and hypoxia release a substantial number of syncytiotrophoblast-derived extracellular vesicles (SDEVs) directly into the maternal circulation (Tannetta et al., 2017a). This review focuses on the biological activities of these SDEVs and their potential roles in the pathogenesis of PE (Table 1).

**TABLE 1:** Analyses of SDEVs in experiments *in vitro* and the pathogenesis of preeclampsia.

**Date**	**Author**	**Samples**	**Test of SDEVs**	**Findings**	**References**
**SDEVs and preeclampsia-associated damage of the vascular endothelium**					
1993	Smárason AK et al.	Placentae of PE and normal pregnancy	BCA	Both SDEVs suppressed human umbilical vein endothelial cell proliferation.	Smarason et al., 1993
1997	Cockell AP et al.	Placenta of normal pregnancy	BCA	SDEVs inhibited endothelial cell-dependent relaxion of small arteries.	Cockell et al., 1997
2000	Kertesz Z et al.	Placenta of normal pregnancy	BCA	Adhesion molecules integrin α_5_ and α_v_ and DPPIV of SDEVs inhibited proliferation of HUVECs.	Kertesz et al., 2000
2005	Gupta AK et al.	Placenta of normal pregnancy	BCA	SDEVs from mechanical dissection, *in vitro* placental explant culture and perfusion of placental cotyledons inhibited HUVEC proliferation	Gupta et al., 2005c
2013	Tannetta et al.	Placentae of PE and normal pregnancy	Flow cytometry Western blotting	For PE placenta, Eng expression of mSDEVs was increased and Eng expression of pSDEVs was decreased. Increased Flt-1/sFlt-1 and decreased PLAP content were observed in PE placenta pSDEVs.	Tannetta et al., 2013
2017	Motta-Mejia C et al.	Placenta of normal pregnancy	Flow cytometry Western blotting	SDEVs isolated from PE perfused placentae had decreased levels of SDEVs-eNOS and decreased NO activity.	Motta-Mejia et al., 2017
**SDEVs and preeclampsia associated with inflammatory responsiveness**					
1999	von Dadelszen P et al.	Placenta of normal pregnancy	/	Supernatants from HUVECs cultured with SDEVs caused significant activation of PBLs (including granulocytes, monocytes, and lymphocytes).	Von Dadelszen et al., 1999
2005	Gupta AK et al.	Placenta of normal pregnancy	BCA	SDEVs activated neutrophils in an independent manner and caused NET formation.	Gupta et al., 2005a
2005	Gupta AK et al.	Placenta of normal pregnancy	BCA	SDEVs significantly induced T cell proliferation.	Gupta et al., 2005b
2007	Germain SJ et al.	Placenta of normal pregnancy	ELISA	SDEVs prepared by pS stimulated PBMCs to produce inflammatory cytokines.	Germain et al., 2007
2010	Messerli M et al.	Placenta of normal pregnancy	/	SDEVs of pS and eS activated peripheral blood monocytes.	Messerli et al., 2010
2012	Holder et al.	Placentae of PE and normal pregnancy	/	SDEVs activated PBMCs, as shown by elevated IL-1B. SDEVs from PE placenta exacerbated the LPS response.	Holder et al., 2012
2012	Lee SM et al.	Trophoblast-derived cell line	FACS	SDEVs from hypoxic trophoblasts stimulated PBMCs to release increased concentrations of IL-6 and TNF-α.	Lee et al., 2012
2014	Joerger-Messerli, M. S. et al.	Placentae of PE and normal pregnancy	BCA	SDEVs stimulated PBMCs to secrete IL-6 and IL-8.	Joerger-Messerli et al., 2014
**STBMV and preeclampsia-associated coagulopathy**					
2011	Guller S et al.	Placenta of normal pregnancy	DC Protein Assay Flow cytometry	Eng and PAI-2 were localized to the surface of placental microvesicles	Guller et al., 2011
2011	Gardiner et al.	Placentae of PE and normal pregnancy	BCA Flow cytometry	SDEVs, especially from PE placenta, triggered thrombin generation in normal plasma in a TF-dependent manner, indicating that TF is expressed by SDEVs.	Gardiner et al., 2011

*BCA, bicinchoninic acid protein assay; DPPIV, dipeptidyl peptidase IV; Eng, endoglin; ELISA, enzyme-linked immunosorbent assays; eNOS, endothelial nitric oxide synthase; eS, in vitro explant cultures; FACS, Fluorescence activated Cell Sorting; Flt-1, fms-like tyrosine kinase 1; HUVEC, human umbilical vein endothelial cell; mSDEVs, SDEVs from mechanical dissection; NETs, neutrophil extracellular traps; NO, nitric oxide; PAI, plasminogen activator inhibitor; PBLs, peripheral blood leukocytes; PBMCs, peripheral blood mononuclear cells; PLAP, placental alkaline phosphatase; pS, placental perfusion; pSDEVs, SDEVs prepared by placental lobe dual perfusion; sFlt-1, soluble fms-like tyrosine kinase 1; TNF-α, tumor necrosis factor-α.*

## Structure and Function of SDEVs

**FIGURE 1:**
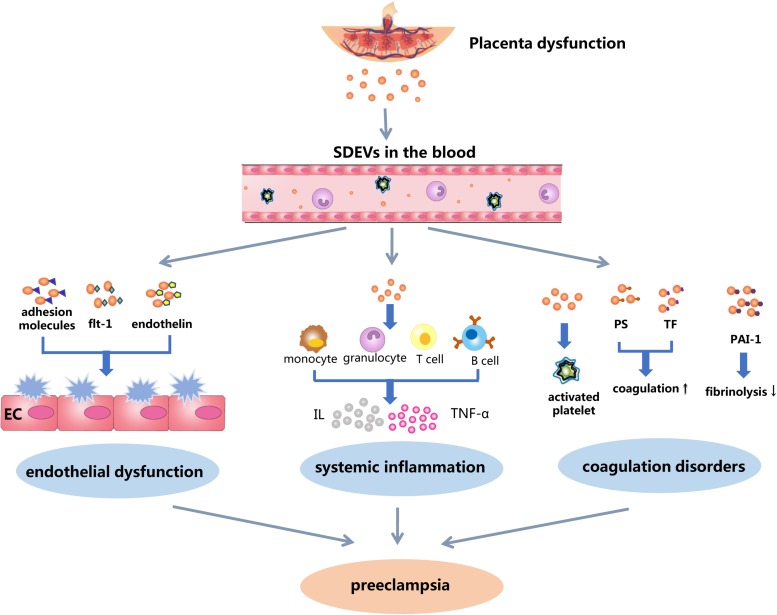
A schematic illustration of SDEVs in the pathophysiology of preeclampsia. Ischemic and hypoxic injures to the placenta induce syncytiotrophoblast cells to undergo apoptosis and release SDEVs into the maternal circulation, where they induce endothelial injury, inflammation, and hypercoagulation. It remains debatable as whether SDEVs from women with normal pregnancies and those from PE patients have similar or distinct structures and biological activities. If it is the former, the same process could occur during normal pregnancies, but at a much lower level. If it is the latter, SDEVs from the two conditions may elicit very distinct systemic responses. In this case, the process depicted here is developed based on research and clinical findings from PE.

SDEVs are alternatively called placental syncytiotrophoblast microvillous membrane (Cockell et al., 1997; Aly et al., 2004), syncytiotrophoblast microparticles (Han et al., 2016) and placental-derived microvesicles (Holder et al., 2012). Similar to EVs of other cells, SDEVs are highly heterogeneous in their morphology, carried cargos, biological properties, and activities toward target cells. Small lipid-bilayer membrane vesicles with diameters of 0.1 to 1 μm are the most common type of SDEVs, but intracellular organelles and granules are also present. These vesicles are derived primarily from apoptotic or activated syncytiotrophoblast cells (Goswami et al., 2006; Reddy et al., 2008) that are directly exposed to the maternal circulation (Chiarello et al., 2018). SDEVs are not simply microvillus membrane fragments but also contain substantial and variable numbers of biologically active molecules that could interact with endothelial cells, platelets, and leukocytes of the maternal circulation. These interactions are important for maternal physiology and maternal-fetal communications during normal pregnancy (Tannetta et al., 2017b). They have physiological immunoregulatory function in the maternal response to pregnancy (Tannetta et al., 2017b; Nair and Salomon, 2018) through the activation of maternal innate immunity, mediation of the maternal systemic inflammatory response, and suppression of immune reactions to the fetus. Smaller and secreted exosomes are also necessary for inducing maternal adaptive changes (Mincheva-Nilsson and Baranov, 2014) and do not appear to cause pathological changes (Rodie et al., 2004). However, membrane SDEVs with surface-exposed anionic phospholipids could induce a hypercoagulable state that is widely reported during normal pregnancy and could be responsible for PE and associated thrombosis. The placenta from PE patients could produce significantly more SDEVs (Tannetta et al., 2017b), whose cargos further exaggerate the maternal response with regard to inflammation, vascular function and coagulation (Figure 1), changing the physiological response of normal pregnancy to a pathological state of preeclampsia. Proinflammatory interleukins (ILs) carried by SDEVs may be responsible for the systemic inflammatory state found in both normal pregnancy and PE, whereas tissue factor (TF) and anionic phosphatidylserine (PS) on the surface of these SDEVs could result in a systemic hypercoagulable state that is found in pregnancy, but significantly enhanced in PE (Gardiner et al., 2011). The adhesion molecules expressed on SDEVs, such as ICAM-1, VCAM-l, E-selectin, F-selectin, and vitronectin mediate the adhesion of SDEVs to target cells and could promote platelet activation and aggregation on the surface of the endothelium. Fms-like tyrosine kinase-1 (Flt-1) [and/or soluble Flt-1 (sFlt-1)] and endothelin found in SDEVs are known to induce endothelial dysfunction (Lok et al., 2008a; Guller et al., 2011). Together, these pathological activities induced by SDEVs are significantly exaggerated in PE to restrict fetal growth and result in recurrent miscarriage (Lok et al., 2011; Baig et al., 2013). Han et al. (2016) recently reported that the RhoB/ROCK-regulated actin arrangement promotes the shedding of SDEVs from syncytiotrophoblasts in PE patients, indicating that regulating RhoB/ROCK signaling may be a new therapeutic strategy for patients with PE.

As their roles in PE are increasingly recognized, the structure and activity of SDEVs have been extensively studied, primarily through *in vitro* experiments. For these studies, samples used for the SDEVs preparation are often generated with one of the four technologies: ➀ mechanical dissection of villous tissue (Smith et al., 1974; Smarason et al., 1993), ➁ villous explant in culture (Huppertz et al., 2003), ➂ perfusion of placental cotyledons (Schneider et al., 1972), and ➃ culture of trophoblast-derived cell lines (Lee et al., 2012). In most studies, EVs thus generated from biological fluids and tissue homogenates are further isolated using differential ultracentrifugation. In addition, density gradient centrifugation, chemical precipitation, particle filtration, size exclusion chromatography, and magnetic bead separation are also used either alone or in different combinations to isolate EVs (Thery and Witwer, 2018). SDEVs thus obtained can be identified, quantified, and characterized using flow cytometry (Marques et al., 2012), enzyme-linked immunosorbent assays (ELISA) (Göhner et al., 2015), electron microscopy (EM), immunoblots and a bicinchoninic acid protein assay (BCA) to quantify membrane protein content (Smarason et al., 1993). More recently, cryo-EM, nanoparticle tracking analysis (NTA), dynamic light scattering, resistive pulse sensing, atomic force microscopy, and Raman spectroscopy have also been used to study SDEVs. However, flow cytometry remains the preferred and most widely used technique for detecting and analyzing EVs (Robert et al., 2012; Van Der Pol et al., 2016). This technology can quantitatively identify EVs by particle size and by specific markers and functional molecules from parental cells (e.g., anionic phospholipids for coagulation). It can also evaluate interactions of EVs with target cells and measure the response from these target cells. In this view, we discuss SDEVs that are identified by the size and expression of placental alkaline phosphatase (PLAP). In addition, standard microbeads are increasingly used to more accurately detect SDEVs of different sizes (Robert et al., 2009). Flow cytometry combined with microbeads allows standardization of detecting of SDEVs and other EVs as markers for clinical diagnosis and outcome predictions in a clinical setting. However, standard flow cytometry may not provide sufficient resolution to detect smaller vesicles of less than 200 nm (e.g., exosomes) (Groot Kormelink et al., 2016; Thery and Witwer, 2018). For these smaller vesicles, high resolution flow cytometry and other techniques are being developed to detect these smaller vesicles in high throughput.

## SDEVs and PE

As pregnancy progresses, trophoblasts undergo repeated cycles of proliferation and apoptosis that are necessary for placental development (Desforges et al., 2013). During the remodeling, apoptotic syncytiotrophoblast cells shed SDEVs into the maternal circulation, leading to progressively increasing numbers of circulating SDEVs, which reach a peak level in the third trimester. However, SDEVs appeared earlier and are at significantly higher levels in patients with PE, primarily because of placental injuries. These SDEVs are also highly heterogeneous in their cells of origin, morphological characteristics, cargo contents, and diverse biological activities. The different levels of SDEVs found in women with normal pregnancy and those with PE raise two important questions. First, do SDEVs produced from the placenta of normal pregnancies differ in structure and activities from those from the placenta of PE patients? If SDEVs produced in the two conditions are identical or similar, what is the threshold level of plasma SDEVs that will trigger PE? Because of lacking standard in sample processing, detecting reagents, and instrument setup, levels of SDEVs found in normal pregnancies and in patients with preeclampsia vary significantly among published reports, but SDEVs have been consistently found higher in PE patients than in women with normal pregnancies (Goswami et al., 2006; Chen Y. et al., 2012). The quantitative difference of plasma SDEVs between PE patients and women with normal pregnancies also varies, ranging from 10 to 250%. However, the fact that SDEVs in PE patients are produced from the placenta exposed to ischemia and hypoxia due to defective trophoblastic invasion (Reddy et al., 2008; Ma et al., 2014) and those during normal pregnancies are produced from placenta development would suggesting that these two types of SDEVs could differ in their biological activities. Second, do SDEVs cause PE or are a result of PE? The answer is likely both because levels of SDEVs in the maternal circulation directly reflect the state of placental injury (Reddy et al., 2008; Chen Y. et al., 2012) and are associated with the severity of hypertension in PE patients (Lok et al., 2008b; Chen Y. et al., 2012). When compared with those from healthy pregnant women, SDEVs from PE patients were reported to have greater proinflammatory (Al-Ofi et al., 2012; Bobek et al., 2015), endothelial activating (Cooper, 1994; Brown et al., 2015), vasoconstrictive (Boisrame-Helms et al., 2015), anti-angiogenic, and procoagulant (Guller et al., 2011; Lok et al., 2011; Rusterholz et al., 2011; Sergeeva et al., 2015) activities. Their plasma levels were closely correlated with the severity of maternal and premature infant complications (Goswami et al., 2006), but definitive evidence remains elusive regarding the PE-causing activity of SDEVs released from injured placenta.

## SDEVs and PE-Associated Inflammation

Pregnancy is not only a state of immune tolerance to the fetus but also a complicated immunomodulatory status that resists the invasion of the potential pathogens. The interactions between the inflammatory response and the immune system at different gestation periods enable the success of the pregnancy (Mor et al., 2011). SDEVs prepared from the normal placenta could stimulate the production of proinflammatory cytokines, such as tumor necrosis factor (TNF)-α, IL-1β, IL-6, IL-8, IL-18, and IL-12 by binding and activating monocytes (Germain et al., 2007; Messerli et al., 2010), contributing to the adoptive changes during normal pregnancy. In contrast, SDEVs from cultured syncytiotrophoblast cells exposed to hypoxia and those from the placenta of PE patients stimulate peripheral monocytes (Messerli et al., 2010), neutrophils, B and T lymphocytes (Gupta et al., 2005b) to release proinflammatory factors, such as IL-1 (Holder et al., 2012), IL-6, IL-8, and TNF-α, resulting in an exaggerated systemic inflammatory response (Gupta et al., 2005a; Germain et al., 2007; Lee et al., 2012). These inflammatory mediators can induce endothelial injuries, leading to tissue edema and vascular leakage (Von Dadelszen et al., 1999). This SDEV-induced systemic inflammation is reported to be inhibited by blocking Toll-like receptor (TLR) signaling or targeting the nuclear factor kappa-light-chain-enhancer of activated B cells (NF-κB) (Joerger-Messerli et al., 2014).

## SDEVs and PE-Associated Endothelial Injury

During normal pregnancy, trophoblasts at the end of their lifespan are shed from the placenta and enter the maternal circulation in a process called trophoblast deportation. These deported trophoblasts and their debris can be trapped in the maternal blood vessels of the lung, but neither clogging of the lung nor induction of the immune response occurs in normal pregnancies. It is believed that endothelial cells phagocytose the deported trophoblasts and apoptosis-derived debris to suppress the activation of endothelial cells caused by the low level of necrotic trophoblastic debris during normal pregnancy (Chen et al., 2006; Chen Q. et al., 2012). When this process becomes dysregulated, the apoptotic trophoblastic debris-mediated protection of endothelial cells is lost and excessive necrotic debris of trophoblasts could then activate endothelial cells or prevent them from self-renewal (Kertesz et al., 2000; Gupta et al., 2005c), consistent with endothelial injury being the pathological hallmark of PE (Alma et al., 2018).

In addition, SDEVs could also stimulate neutrophils to produce superoxide free radicals that further injure endothelial cells in not only placental vasculature but also vessels in other organs (Aly et al., 2004; Xiao et al., 2017). The tyrosine kinase sFlt-1, which is a known anti-angiogenic factor (Ikeda et al., 2011), is expressed on SDEVs and may be responsible for SDEV-induced endothelial injury in patients with PE (Lok et al., 2008a; Tannetta et al., 2013). Furthermore, SDEVs from PE patients or those obtained by the perfusion of placentae from PE patients reduce the production of nitric oxide (NO) from endothelial cells by blocking the synthesis of endothelial nitric oxide synthase (eNOS), contributing to the vascular endothelial dysfunction, hypertension, and proteinuria found in PE patients (Motta-Mejia et al., 2017). In addition to these *in vitro* findings, *ex vivo* experiments further show that, when perfused into subcutaneous adipose arteries obtained from normotensive pregnant women, SDEVs generated from mechanically injured normal placenta induced endothelial dysfunction, as shown by the reduction of acetylcholine release (Smarason et al., 1993; Cockell et al., 1997). Recently, we have shown that pregnant mice infused with SDEVs produced from injured placenta developed a preeclampsia-like phenotype (i.e., hypertension and proteinuria) (Han et al., 2019). The causal activity of SDEVs for PE is further demonstrated in experiments where SDEVs induced the same PE-like phenotype in non-pregnant mice and mice deficient in EV clearance developed spontaneous PE during pregnancy without infusion of SDEVs. Together, these results demonstrate that SDEVs cause endothelial cell injury of the maternal vasculature, but additional studies are required to determine whether SDEVs from PE patients and those with a normal pregnancy have different biological activities due to their diverse expression of surface molecules and cargo contents.

## SDEVs and PE-Associated Coagulation Dysregulation

Pregnant women often develop a hypercoagulable state, especially during the late stage of pregnancy (Stirling et al., 1984). This hypercoagulable state is much more severe in PE patients (He et al., 1997), often resulting in microvascular thrombosis, terminal organ ischemia, and, in worst cases, disseminated intravascular coagulation (Cunningham and Nelson, 2015). This hypercoagulable state could result in extensive deposition of the coagulation product fibrin in the vascular endothelium, furtherdamaging the endothelial function and increasing the rigidity of the vessel wall, which contributes to the development of hypertension (Konukoglu and Uzun, 2017). This pregnancy-induced hypercoagulable state is believed to be caused by placenta-derived procoagulant molecules such as TF and anionic phospholipids such as PS (Lanir et al., 2003; Teng et al., 2010; Han et al., 2019). TF is a transmembrane protein that initiates extrinsic coagulation during hemostasis at the site of vascular injury. This protein is also expressed on the surface of syncytiotrophoblasts, and its expression is increased during placental hypoxia and reoxygenation (Teng et al., 2009). TF is present on the surface of SDEVs, especially those from PE patients (Aharon et al., 2004; Gardiner et al., 2011), indicating that SDEVs are capable of initiating extrinsic coagulation on their surface or in plasma. In addition, PS is also exposed on SDEVs and is critical for accelerating and enhancing coagulation (Owens and Mackman, 2011). The PS externalization occurs on activated or apoptotic syncytiotrophoblast cells (Mooberry and Key, 2016; Vallier et al., 2017). TF and PS exposed on SDEVs are therefore major factors that induce the systemic hypercoagulable state found in patients with PE. This TF- and PS-driven hypercoagulable state was further enhanced by the overexpression of the serine protease plasminogen activator inhibitors-1 (PAI-1) on SDEVs (Guller et al., 2011). PAI-1 inhibits tissue plasminogen activator (tPA) and urokinase plasminogen activator (uPA) that activate plasminogen to trigger clot fibrinolysis and to re-establish blood flow of occluded vessels. PAI-1 expressed on SDEVs could block or reduce fibrinolysis to prevent or delay tissue reperfusion, thus propagating the PE-induced hypercoagulable and prothrombotic state. The placenta also secretes PAI-2, which is found at a high level in the peripheral blood during pregnancy and is involved in the invasion and remodeling of fetal and uterine tissues, but its role in the development of PE and the associated hypercoagulable state remains unknown (Astedt et al., 1998). A recent study showed a significant increase in protein tyrosine phosphorylation in platelets stimulated by SDEVs purified from PE patients but not those from women with normal pregnancies (Tannetta et al., 2015), suggesting that SDEVs from the injured placenta could activate platelets. This SDEV-induced platelet activation enhances the hypercoagulable state to increase the risk of thrombosis in patients with PE (Tannetta et al., 2015). Consistent with this platelet role in PE, the platelet antagonist aspirin blocks the effect of SDEVs on platelet aggregation *in vitro* (Tannetta et al., 2015) and improves maternal and fetal outcomes of PE patients (ACOG, 2018).

## Conclusion

Together, the available clinical and laboratory data suggest that PE can be induced by increasing the level and activity of SDEVs in the maternal circulation. The elevated levels of circulating SDEVs are caused not only by excessive production from diseased placenta but also by insufficient scavenging of these SDEVs from circulation through the intrinsic scavenging system (Kaminska et al., 2018). This scavenging system could be overwhelmed by the high levels of SDEVs released from apoptotic syncytiotrophoblast cells (Knight et al., 1998), resulting in a consumptive scavenging deficiency. There are considerable interests in identifying early predictive markers for preeclampsia, which often develops during the late stage of pregnancy. SDEVs are an ideal candidate. Characterizing SDEVs in peripheral blood samples may provide new biomarkers for clinical prediction and early diagnosis of PE, but its clinical application has not been widely adapted in clinical setting and would require the standardization of blood sample collecting and processing, detection reagents and instrument setup. Reducing SDEVs release and increasing their clearance may be new therapeutic strategies for the treatment of PE.

## Author Contributions

CH, LH, PH, and YC conducted the literature search and wrote the manuscript. YW and FX wrote the manuscript.

## Conflict of Interest

The authors declare that the research was conducted in the absence of any commercial or financial relationships that could be construed as a potential conflict of interest.
